# 

**Published:** 1893-11

**Authors:** 


### CONTENTS:

ORIGINAL ARTICLES: PAGE

The Hackney Horse. By R. S. Huidekoper, M.D., Vet. .......... 281

New Method of Treating Ophthalmia. By Dr. R. H. Harrison .......... 306

Scouring in Calves. By T. S. Gold .......... 312

Millet Disease. By T. D. Hinebauch, M.S., V.S .......... 313

Actinomycosis Bovis, or "Lump Jaw." By Prof. N. S. Mayo, D.V.S .......... 322

Fistula. By Prof. M. H. Reynolds, M.D., V.M .......... 323

EDITORIAL:

United States Veterinary Medical Association .......... 342

SOCIETY PROCEEDINGS:

New York State Veterinary Medical Society. Fourth Semi-Annual Meeting .......... 345
Ohio State Veterinary Medical Association. Tenth Semi-Annual Meeting .......... 361
Indiana Association of Veterinary Graduates. Semi-Annual Meeting .......... 362

Army Legislation .......... 364

